# Sturdy Positioning with High Sensitivity GPS Sensors Under Adverse Conditions

**DOI:** 10.3390/s100908332

**Published:** 2010-09-03

**Authors:** Klemen Kozmus Trajkovski, Oskar Sterle, Bojan Stopar

**Affiliations:** University of Ljubljana, Faculty of Civil and Geodetic Engineering, Jamova 2, SI-1000 Ljubljana, Slovenia; E-Mails: oskar.sterle@fgg.uni-lj.si (O.S.); bojan.stopar@fgg.uni-lj.si (B.S.)

**Keywords:** GNSS, GPS positioning, high sensitivity, differential GPS, adverse conditions, Doppler observations

## Abstract

High sensitivity GPS receivers have extended the use of GNSS navigation to environments which were previously deemed unsuitable for satellite signal reception. Under adverse conditions the signals become attenuated and reflected. High sensitivity receivers achieve signal reception by using a large number of correlators and an extended integration time. Processing the observation data in dynamic and rapidly changing conditions requires a careful and consistent treatment. Code-based autonomous solutions can cause major errors in the estimated position, due primarily to multipath effects. A custom procedure of autonomous GPS positioning has been developed, boosting the positioning performance through appropriate processing of code and Doppler observations. Besides the common positioning procedures, robust estimation methods have been used to minimise the effects of gross observation errors. In normal conditions, differential GNSS yields good results, however, under adverse conditions, it fails to improve significantly the receiver’s position. Therefore, a so-called conditional DGPS has been developed which determines the position differentially by using data from the strong signals only. These custom-developed procedures have been tested in different conditions in static and kinematic cases and the results have been compared to those processed by the receiver.

## 1. Introduction

Standard GNSS positioning was previously limited to open areas that guaranteed the unobstructed reception of GNSS satellites signals. With the emergence of so-called high sensitivity GPS receivers (HS GPS), the practical application of GNSS has expanded to include areas with poor signal reception. Challenging environments, such as urban canyons, forested areas and indoors are often labelled as “indoor” environments [1]. Such environments cause signal attenuation and signal reflection from nearby reflective surfaces, resulting in multipath errors. Signal attenuation and multipath can cause large errors in the position estimation.

Upon its arrival on Earth, the GPS signal is already weak. Regular GNSS receivers are able to track signals attenuated by the atmosphere only. If a signal passes through a physical substance, the signal becomes too weak to be tracked by a regular GNSS receiver. HS GPS receivers are able to track exceptionally weak signals by incorporating a large number of correlators and employing an extended integration period. Regular GNSS receivers incorporate up to 50 correlators [2], whereas the latest HS GPS chips incorporate more than one million correlators, e.g., u-blox 5 and u-blox 6.The typical integration time for regular GNSS receivers is a few ms [1], while HS GPS receivers perform integration over a longer interval. Integration can be extended to several hundred ms [1].

According to [3], the tracking performance does not entirely depend on the signal strength but also on the signal-to-noise ratio (SNR) and more specifically on the SNR in a 1 Hz bandwidth, *i.e.*, the carrier-to-noise density ratio (C/N_0_). Regular GPS receivers are able to acquire and track signals with a C/N_0_ above 33–35 dBHz. The HS GPS receivers in our experiments were able to track signals with a C/N_0_ as low as 10 dBHz.

HS GPS receivers track signals from GPS satellites on the L_1_ frequency. The results of the signal tracking are usually code pseudoranges, carrier-phase and Doppler observables. A SNR value is also typically available. Most of the HS GPS receivers available on the open market determine the current position internally and output their results via NMEA 0183 messages. Only a few of them can be setup to output raw observation data.

## 2. Development of the Positioning Procedure

Custom positioning procedures can be developed only by processing raw observation data. Therefore, only GPS receivers with raw data output were considered for the survey tests. Two u-blox evaluation kits with a raw data output option were used in the tests, namely the AEK-4T and EVK-5T. The AEK-4T employs the Antaris GPS chip and offers rates of up to 10 Hz for raw data, whilst the EVK-5T relies on the u-blox 5 Positioning Engine which supports a 2 Hz raw data update rate. Both receivers are able to determine and output the following data: code pseudoranges; carrier-phase measurements; Doppler measurements and SNR values. However, the AEK-4T receiver is able to output carrier-phase observables only for signals with a SNR over 30 dBHz, meaning that there is no carrier-phase data for the attenuated signals. On the other hand, carrier-phase measurements from the EVK-5T are available even for the weakest signals. Owing to the limitations of one of the receivers, the custom positioning solution depends on code pseudoranges and Doppler observables only.

Basic autonomous positioning is based on processing the code pseudoranges in the least squares adjustment. Four unknowns have to be determined in the process: the 3D position coordinates of the receiver and the receiver clock offset. A pseudorange is affected by a number of biases and possible errors: the satellite position; the satellite clock offsets; the ionosphere bias; the troposphere bias; multipath and noise. All biases have to be modelled to obtain the estimated unknowns to the highest possible degree of accuracy and reliability.

Multipath is a phenomenon whereby the receiver’s antenna receives a GPS signal from different paths. This is caused by the reflection of the signal off reflective surfaces near the receiver. Multipath depends entirely on the local environment and is therefore nearly impossible to model. Theoretically, multipath can reach up to one half of a wavelength of a pseudorange (up to 150 m for code observations) and up to one quarter of a wavelength of the carrier phase (a few cm) [4]. Since Doppler measurements are affected by the bias rates only [5], the multipath effect on Doppler observations should be on the level of the carrier phase multipath effects. Multipath is undoubtedly the most significant bias in code pseudoranges observed in “indoor” positioning.

Atmospheric biases can be mitigated with ionosphere and troposphere models. An ionosphere bias can be cancelled out by applying dual frequency observations. However, HS GPS receivers are single frequency receivers and for such observations an ionosphere model has to be used, e.g., the Klobuchar model. A troposphere bias can be mitigated via a combination of a tropospheric zenith delay and a mapping function. More on the topic can be found in [4].

Some additional methods are used in classical GPS positioning procedures to attempt to enhance the accuracy of the results achieved. Signals with low elevation angles are affected to a greater degree with atmospheric refraction than signals at high elevation angles. Elevation angle weighting can be used to compensate for the different quality of the signals. Signals with lower SNR values are generally assumed to be of a lesser quality than those with high SNR values. Some authors suggest using SNR weighting [6].

If carrier-phase or Doppler observations are available, then errors in code-based observations can be mitigated by smoothing code-based observations. This smoothing technique is achieved using aso-called Hatch filter [7].

Outliers are common in observations that take place in multipath and attenuated environments. The first option is to try to eliminate outliers from the mathematical model. However, based on our experience, it is difficult to accurately detect and eliminate outliers in such conditions. The second option is to minimise the effect of gross errors on the final solution. This can be achieved by robust estimation.

Least squares adjustment methods yield a correct interpretation of the results if no outliers are present in the solution and all the systematic errors are modelled. According to [8,9], robust estimation methods perform considerably better than least squares when gross errors are present in the mathematical model. One of the most widely used robust estimation methods is the L_1_-norm.The weight function of the L_1_-norm is defined as:

(1) $$w_{i}=\frac{1}{\left|v_{i}\right|}$$

where *v_i_* is the residual of the *i*-th observation and *w_i_* is the weight of the *i*-th observation in the next iteration. The residual vector *v* is obtained during the least squares adjustment procedure and the residual value *v_i_* represents the difference between the input value and the adjusted value of the observation. The robust estimation procedure relies on the basic least squares equations with the additional observation weighting.

### 2.1. Custom Positioning Procedure

The custom developed procedure for GNSS positioning under adverse conditions relies on the modified L_1_-norm robust estimation. A residual value of zero results in a deletion by zero, see Equation (1). To avoid such a result, each observation with a residual of less than 10^−4^ m is given a weight of 10^4^. The modified L_1_-norm weight function is therefore defined as:

(2) $$w_{i}=\left\{ \begin{array}{ll} \frac{1}{\left|v_{i}\right|} & \left|v_{i}\right|\geq 10^{-4}m\\ 10^{4} & \left|v_{i}\right|<10^{-4}m \end{array}\right.$$

As mentioned above, Doppler measurements can be used to smooth code observations. In our experiments, however, this procedure has not produced promising results. Doppler values can also be used for so-called Doppler positioning. Positioning is realised using Doppler observations only, as suggested in [10], but practical realisation is particularly difficult to achieve in environments with a decreased number of available signals. Doppler measurements basically measure the range change between a receiver and a satellite between consecutive epochs. The following equation can be used to calculate the delta range [11]:

(3) $$\Delta D=\frac{1}{2}\left(D1_{i-1}+D1_{i}\right)\cdot \lambda \cdot \mathrm{dt}$$

where *D*1_*i*−1_ and *D*1_*i*_ are Doppler observables in the previous and the current epochs, respectively, *λ* is the wavelength of GPS L_1_frequency and *dt* is the time interval between consecutive epochs. However, this method of Doppler positioning requires reference range values. These can only be achieved using code pseudoranges. Therefore, the initial position can only be determined by code-based observations. This would ideally be determined in an environment with little or no multipath or attenuation. Differential GPS (DGPS) can also be used in such environments if the reference station data is available. DGPS increases the accuracy of the initial positions and consequentially all the following positions if determined relatively by Doppler positioning.

The custom procedure for the positioning of the following epochs (after the initial epoch) determines the receiver clock offset and the position unknowns separately. The receiver clock offset is determined by Doppler observables only. The use of code-based observations in estimating the receiver clock offset can cause anomalies which often occur when the number of tracked satellites changes between epochs. After the receiver clock offset has been determined, the position unknowns are determined using code and Doppler observations. Doppler observations are included in the model as shown in Equation (3). Both types of observation are equally weighted.

One additional feature of the procedure is the recalculation of the ranges after each epoch. The ranges between the receiver and the satellites are calculated using the adjusted current position. These ranges then become input data for the following epoch instead of the pseudoranges.

### 2.2. DGPS and Conditional DGPS

DGPS is a positioning technique whereby the position of the rover is determined using code pseudorange corrections, calculated by the reference receiver. The achieved position accuracy is refined to a scale of 1 metre, whereas the accuracy of an autonomous position is around 10 metres [12]. DGPS assumes good operational conditions in the proximity of both receivers. DGPS does not function well under adverse conditions, because the noise and multipath are amplified in the differencing process [12].

**Figure 1.**
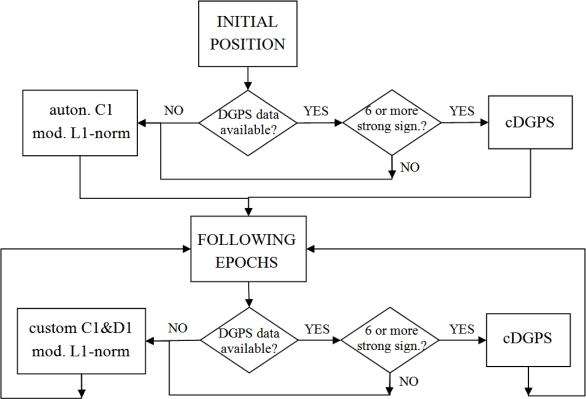
Flowchart of the custom positioning procedures.

Since DGPS does not perform well under adverse conditions where signal reception is poor, so-called conditional DGPS (cDGPS) has been developed, in which only strong signals are used to secure the positioning. Strong signals are defined as those with a SNR value above 35 dBHz, which is usually the limit value for the signal tracking of regular GNSS receivers [3].The cDGPS is performed in each epoch with 6 or more strong signals which are also tracked by the reference receiver. If the cDGPS cannot be performed, the positions are determined by the custom autonomous positioning procedure. Another useful feature of the cDGPS is its ability to calculate residuals for “no cDGPS” epochs. At the epoch the conditional DGPS is viable again, the position is determined by the cDGPS and by the custom procedure. The difference between both positions is time-related distributed back to “no cDGPS” epochs. This feature has no bearing on real-time surveys. Nevertheless, it is useful for a post-analysis of the calculated positions. It should be noted that the cDGPS can only be performed when reference station data is available. Figure 1 displays the basic data flow of the custom developed autonomous and cDGPS positioning procedures.

## 3. Experiments, Results and Discussion

**Figure 2.**
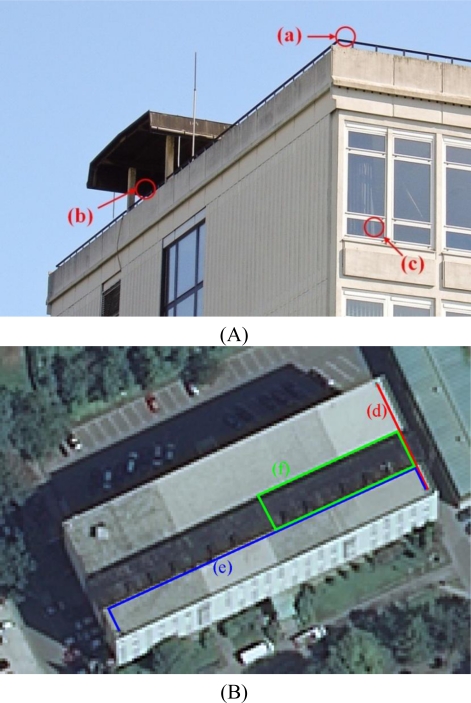
**(A)** Reference points: (a) normal conditions; (b) adverse conditions; (c) indoors. **(B)** Kinematic survey outlines on an orthophoto image: (d) mixed conditions; (e) mixed conditions; (f) adverse conditions.

The experiments were carried out on-site at the Faculty of Civil and Geodetic Engineering in Ljubljana, Slovenia. The tests were performed under different conditions and at different times in static and kinematic modes. Reference points and kinematic survey outlines are shown in Figure 2 (A). The reference point positions were determined by precise GNSS and total station measurements. An extending roof turret above the rooftop of the building (partially visible in Figure 2 (A) and represented by the dark shape in Figure 2 (B)) was used to simulate adverse conditions, where the signals become attenuated and reflected.

The horizontal coordinates of the surveyed points are expressed as *N* and *E* in a projection plane, while the vertical component is expressed as the height above ellipsoid *h*. The observables used in the positioning procedure are labelled as in RINEX files: *C1* code pseudoranges and *D1* Doppler values. All tests were carried out and processed in a 1 Hz kinematic mode.

Several autonomous solutions were determined from each survey: C1 (code-based positioning); D1 (Doppler positioning); C1&D1 (common processing of code and Doppler observations); custom C1&D1 (as described in Section 2.1.); NMEA (positions, determined by the receiver, extracted from NMEA messages). The elevation angle weighting and SNR weighting were also tested. DGPS solutions are labelled DGPS, whereas the conditional DGPS is represented by the label cDGPS. The symbols I and T represent the use of ionosphere and troposphere models respectively.

### 3.1. DGPS Under Adverse Conditions

**Figure 3.**
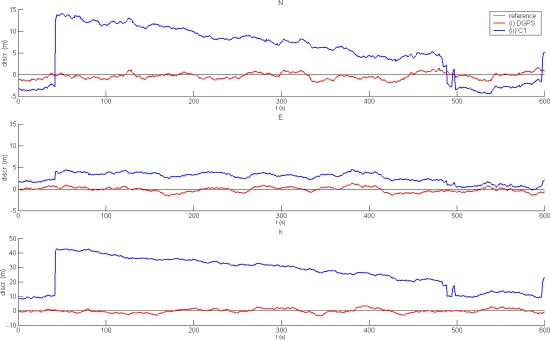
Performance of DGPS under normal conditions: (i) DGPS positioning; (ii) code-based autonomous positioning.

The first task was to assess DGPS under different conditions. As expected, DGPS performs well under normal conditions. The results of a 10-minute static survey in environment (a) are shown in Figure 3. The DGPS positions are compared to the basic positioning mode using code pseudoranges only. The graphs illustrate discrepancies in the solutions from the reference values. Basic statistics of both solutions are listed in Table 1.

**Figure 4.**
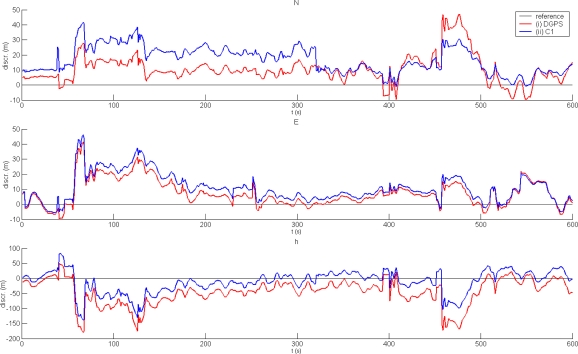
Performance of DGPS under adverse conditions: (i) DGPS positioning; (ii) code-based autonomous positioning.

Under adverse conditions, the results are markedly different. Figure 4 illustrates the results of DGPS and basic positioning in environment (b). The results of DGPS are similar to or worse than those achieved by basic positioning. Basic statistics of both solutions are shown in Table 2.

### 3.2. Indoor Positioning

**Figure 5.**
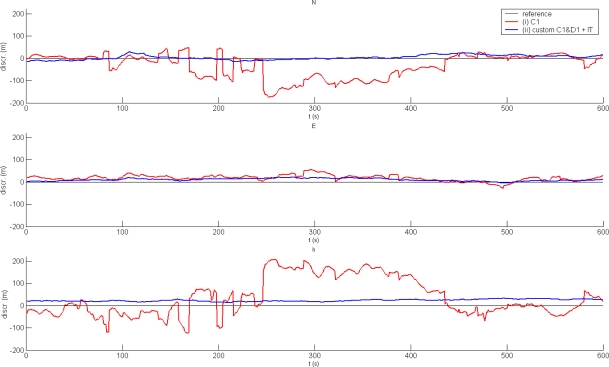
The best and worst indoor solutions: (i) autonomous C1 positioning; (ii) custom positioning procedure with C1&D1, plus ionosphere and troposphere models.

All signals are attenuated and multipath becomes a permanent feature inside objects. This causes large errors in positions which were estimated using code-based positioning. As seen in Figure 5, the discrepancies in reference position exceed 100 m in a single component in environment (c).The best solution is also illustrated by way of comparison. The best solution is the custom procedure using C1 and D1 with the application of the Klobuchar ionosphere model and the troposphere model, consisting of the Hopfield zenith delay estimation and the Niell mapping function. Basic statistics of both solutions are listed in Table 3.

### 3.3. Kinematic Surveys Under Adverse Conditions

**Figure 6.**
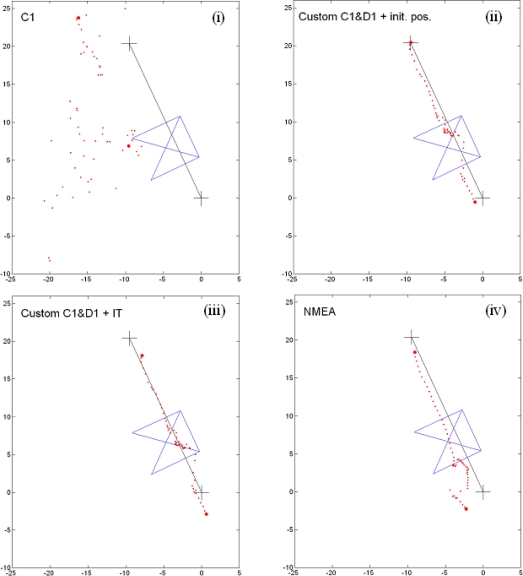
Solutions in a mixed-condition environment (d): **(i)** autonomous C1 positioning; **(ii)** custom positioning procedure with C1&D1, initial position presumed known; **(iii)** custom positioning procedure with C1&D1, plus ionosphere and troposphere models; **(iv)** internal positioning of the receiver. Units are in meters.

The positioning procedures and the equipment were both tested in different kinematic surveys and under different conditions. Their outlines are depicted in Figure 2 (B). Environment (d) was a mixed condition environment. The start and the end of the survey represented relatively normal conditions, however, in the middle, the survey stops underneath the overhanging roof turret for approximately 30 seconds. The whole survey takes 60 seconds. Some of the results of the developed solutions are shown in Figure 6. The black line represents the approximate trajectory and the blue outline depicts part of the overhanging roof. The first and the last determined positions are marked with larger dots. The positions are shown in the projection plane. Plotted units are in metres.

Once again, code-only positioning yields major position errors—see plot (i). The results are considerably better using the custom positioning procedure, when either the initial position is known (ii) or the ionosphere and the troposphere models are used (iii). As a comparison, the internal position is also shown—see plot (iv). The positions remain close to one another, although the cluster of points ought to be under the overhanging roof.

**Figure 7.**
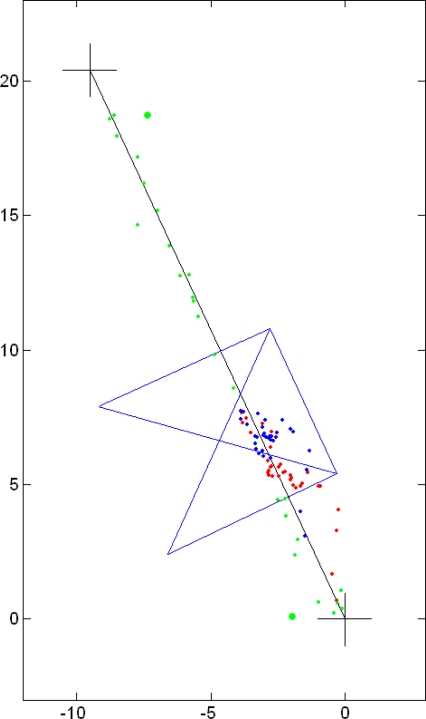
Conditional DGPS in environment (d): ( 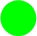) cDGPS positions; ( 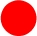) custom positioning procedure with C1&D1; ( 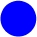) recalculated positions.Units are in meters.

The results of the cDGPS of the same survey are shown in Figure 7. Whenever possible, the cDGPS was performed (green dots). When this was not possible, the custom autonomous positioning was used (red dots). The recalculation of the “no cDGPS” positions was also applied (blue dots).

**Figure 8.**
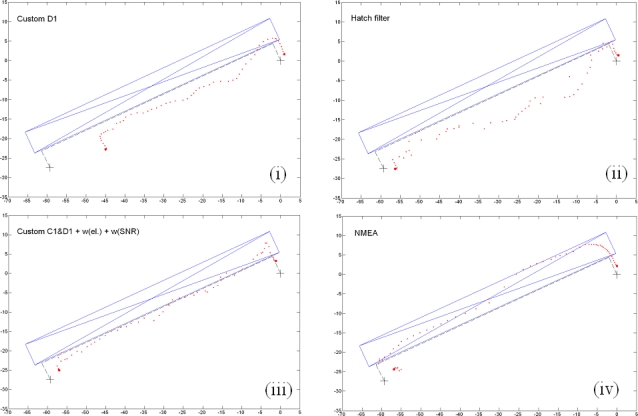
Solutions in a mixed-condition environment (e): **(i)** autonomous Doppler positioning with custom procedure; **(ii)** Doppler smoothing of code observations with the Hatch filter; **(iii)** custom positioning procedure with C1&D1, plus elevation angle weighting and SNR weighting; **(iv)** internal positioning of the receiver. Units are in meters.

Figure 8 displays some of the characteristic solutions of environment (e). Most of the survey takes place under adverse conditions beneath the roof gutter. The survey path is denoted by the dashed line. The blue outline depicts the overhanging roof.

The first plot (i) demonstrates why the Doppler-only solution is not the best option. Whilst the Doppler positioning performs particularly well under normal conditions this is not the case under adverse conditions. The path scale appears to be slightly reduced. Plot (ii) illustrates the results of Doppler smoothing of code observations using the Hatch filter. The procedure causes major deviations from the actual path. In this particular case, the best solution is the custom procedure of C1 and D1 with the additional application of the elevation angle weighting and the SNR weighting. Again, the results of the internal processing are also shown as a comparison.

**Figure 9.**
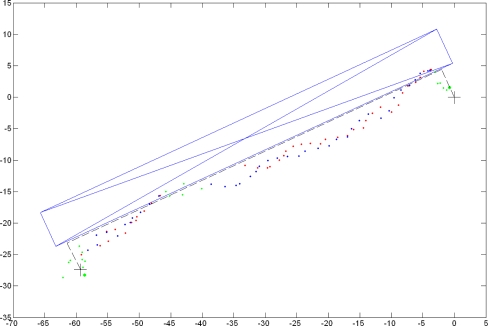
Conditional DGPS inenvironment (e): ( 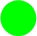) cDGPS positions; ( 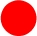) custom positioning procedure with C1&D1; ( 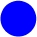) recalculated positions. Units are in meters.

The results of the cDGPS of the same survey are shown in Figure 9. The cDGPS is active at the start and at the end of the survey as well as during 5 epochs in between (see green dots). This solution represents the best of all outcomes, especially when the recalculated positions are considered (blue dots instead of red dots).

**Figure 10.**
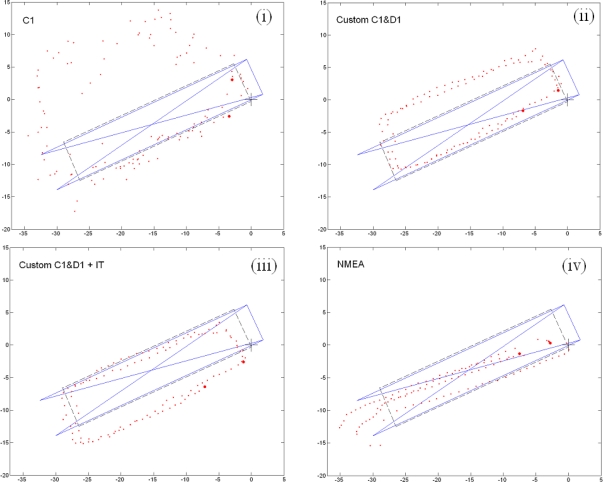
Solutions under adverse conditions (f): **(i)** autonomous code positioning; **(ii)** custom positioning procedure with C1&D1; **(iii)** custom positioning procedure with C1&D1, plus ionosphere and troposphere models; **(iv)** internal positioning of the receiver. Units are in meters.

Environment (f) presents consistently adverse conditions for the reception of GPS signals. Some characteristic solutions are presented in Figure 10. The dashed line represents the path travelled, while the blue outline depicts part of the roof overhang. The survey included two 60 second rounds.

Once again, the custom positioning procedure offers the best results. If the atmosphere models are applied, the positions shift by approximately 4 metres to the south, although the heights are considerably closer to the real values. The difference in the height component is more than 30 metres. If the elevation angle weighting is applied, the positions shift almost 20 metres to the north. The cDGPS cannot be performed since only 1 epoch fulfils the required conditions for cDGPS.

### 3.4. Summary of Results

A wide range of experiments under different conditions were carried out. Several solutions were obtained for each test survey, although only some of the solutions for each case have been presented. Other solutions and cases not presented here demonstrated the same tendencies.

In general, basic positioning using code pseudoranges only tend to cause major position errors beyond 100 m under adverse conditions. The application of Doppler observations in addition to code-based observations and proper processing procedure can drastically improve the accuracy of the results achieved. The Doppler-only solution is clearly deficient, as can be seen in Figure 8. The smoothing of code-based observations with Doppler observations using the Hatch filter does not perform effectively under such conditions either, as Figure 8 illustrates. Although the use of the elevation angle weighting and the SNR weighting has the potential to improve the quality of the results (see Figure 8), the use of additional weighting proved risky, since it can also produce poor results.

The best overall solution and also the sturdiest positioning technique is the custom procedure using both types of observables, code and Doppler. As can be seen from Figures 7 to 11, the quality of the calculated positions is considerably higher in comparison to the basic positioning. The accuracy of the positions falls within a reasonable value range, *i.e.*, 10–20 metres, even under particularly adverse conditions. It should be noted that the positioning mode is still autonomous. The results achieved proved to be better than the results of the internally derived positions from the receiver (see Figures 6, 8 and 10).

The ionosphere and the troposphere both affect the propagation of all signals, irrespective of the conditions experienced by the receiver. Therefore, the ionosphere and the troposphere models should be used in any case to enhance the accuracy of the results. Proof of this is indicated by the case studies presented.

The conditional DGPS is applicable in a mixed-condition environment of a kinematic survey. If the conditions for the reception of the signals are particularly bad, then the cDGPS cannot be used. Conditional DGPS can be used however under conditions which experience periods of minor attenuation and multipath level. Results of the cDGPS in two cases can be seen in Figure 7 and Figure 9. The results are noticeably improved following recalculation of the positions.

## 4. Conclusions

High sensitivity GPS extends the use of GNSS positioning to environments where signals are attenuated and reflected. HS GPS enables positioning even indoors, although only to a certain degree. GPS positioning inside objects is usually limited to top floors and the proximity of outer walls.

Code-based autonomous positioning can often result in major errors of the estimated position. Therefore, observations under adverse conditions should be treated with caution. Owing to the limitations of the equipment available, the custom developed positioning is based on single frequency code pseudoranges and Doppler observations. The procedure relies on the modified L_1_-norm robust estimation to mitigate the influence of gross observation errors. The receiver clock offset and the position unknowns are determined separately. The clock offset is estimated using Doppler observations only, whereas the position is determined using both code and Doppler observables. An additional feature is the recalculation of the satellite ranges after each epoch. Geometric ranges are used instead of pseudoranges in the subsequent epoch. The initial position, however, has to be determined by code-only positioning since only relative velocities between the receiver and the satellites can be derived from Doppler observations. Therefore, in order to consider Doppler observables for positioning, reference ranges are required. The accuracy of the positions of the subsequent epochs depends upon the accuracy of the initial position. For this reason, it is essential that the initial position should be determined in an environment where conditions are favourable. The best initial position would ideally be determined by DGPS or by the cDGPS.

Additional models and weightings can be applied to the positioning procedure. The elevation angle weighting and the SNR weighting can, in some cases, result in a better solution, although they can also cause gross position errors. On the other hand, the ionosphere and the troposphere models should be used in any case and under any conditions. The custom positioning procedure using code and Doppler observations with applied atmosphere models offers the best results for autonomous positioning under adverse conditions. The accuracy achieved is close to the accuracy level obtained under normal conditions.

Under adverse conditions DGPS cannot be used because the quality of the results deteriorates. On the other hand, conditional DGPS maybe applicable if enough strong signals are available. In should be noted that DGPS in general can only be performed if the data from a reference receiver is available. The use of conditional DGPS raises the quality of the calculated positions considerably.

## Figures and Tables

**Table 1.** Statisticsof autonomous and DGPS solutions under normal conditions.

	**C1**			**DGPS**		
	**N**	**E**	**h**	**N**	**E**	**h**
min (m)	−4.4	−0.2	7.9	−1.9	−1.6	−3.7
max (m)	14.2	4.5	43.0	1.5	1.3	3.2
mean (m)	5.4	2.7	26.1	−0.3	−0.1	−0.2
st.dev. (m)	5.6	1.1	10.5	0.7	0.6	1.4

**Table 2.** Statistics of autonomous and DGPS solutions under adverse conditions.

	**C1**			**DGPS**		
	**N**	**E**	**h**	**N**	**E**	**h**
min (m)	−1.0	−5.7	−138.2	−9.5	−9.5	−179.2
max (m)	41.5	46.1	82.5	47.0	41.4	49.7
mean (m)	16.8	10.8	−11.9	10.5	7.5	−45.2
st.dev. (m)	8.9	9.3	37.3	9.4	8.7	43.4

**Table 3.** Statistics of the worst and best indoor solutions.

	**C1**			**custom**		
	**N**	**E**	**h**	**N**	**E**	**h**
min (m)	−175.8	−29.3	−124.0	−14.5	−5.4	12.9
max (m)	49.1	55.5	206.2	29.0	21.4	32.4
mean (m)	−34.0	17.0	36.4	3.5	9.3	22.7
st.dev. (m)	53.2	13.3	84.8	10.5	6.0	4.8
